# Hydroquinone Exhibits In Vitro and In Vivo Anti-Cancer Activity in Cancer Cells and Mice

**DOI:** 10.3390/ijms19030903

**Published:** 2018-03-19

**Authors:** Se Eun Byeon, Young-Su Yi, Jongsung Lee, Woo Seok Yang, Ji Hye Kim, Jooyoung Kim, Suntaek Hong, Jong-Hoon Kim, Jae Youl Cho

**Affiliations:** 1 Department of Genetic Engineering, Sungkyunkwan University, Suwon 16419, Korea; gyo8467@gmail.com (S.E.B.); bioneer@skku.edu (J.L.); real0902@gmail.com (W.S.Y.); kjhmlkjhml@hanmail.net (J.H.K.); 2 Department of Pharmaceutical Engineering, Cheongju University, Cheongju 28503, Korea; 62fdc@hanmail.net; 3 Department of Biochemistry, Lee Gil Ya Cancer and Diabetes Institute, Gachon University, Incheon 21999, Korea; ruffini@naver.com (J.K.); sthong@gachon.ac.kr (S.H.); 4 Department of Physiology, College of Veterinary Medicine, Chonbuk National University, Iksan 54596, Korea

**Keywords:** hydroquinone, cytotoxicity, anti-angiogenesis, anti-metastasis, anti-tumorigenic response

## Abstract

Hydroquinone (HQ, 1,4-benzenediol) is a hydroxylated benzene metabolite with various biological activities, including anti-oxidative, neuroprotective, immunomodulatory, and anti-inflammatory functions. However, the anti-cancer activity of HQ is not well understood. In this study, the in vitro and in vivo anti-cancer activity of HQ was investigated in various cancer cells and tumor-bearing mouse models. HQ significantly induced the death of A431, SYF, B16F10, and MDA-MB-231 cells and also showed a synergistic effect on A431 cell death with other anti-cancer agents, such as adenosine-2′,3′-dialdehyde and buthionine sulfoximine. In addition, HQ suppressed angiogenesis in fertilized chicken embryos. Moreover, HQ prevented lung metastasis of melanoma cells in mice in a dose-dependent manner without toxicity and adverse effects. HQ (10 mg/kg) also suppressed the generation of colon and reduced the thickness of colon tissues in azoxymethane/dextran sodium sulfate-injected mice. This study strongly suggests that HQ possesses in vitro and in vivo anti-cancer activity and provides evidence that HQ could be developed as an effective and safe anti-cancer drug.

## 1. Introduction

Cancer is one of the greatest threats to human health worldwide and the second most common cause of death. Approximately, 7.6 million people die from cancer annually and this number is expected to increase to around 13.1 million by 2030 [1]. Cancer is a multifactorial and complicated disease. Biological systems have evolved various mechanisms to suppress cancer proliferation, including cell cycle checkpoints, tumor suppressor genes, and DNA repair machinery. Several studies have demonstrated effective ways to prevent and treat cancers, which have led to the development of methods to diagnose cancers as well as a variety of drugs to treat cancers. Although many cancer drugs have been actively developed and approved, they have variable ranges of side effects and toxicities, emphasizing the need for the development of more effective and promising cancer drugs with minimal adverse effects. In fact, naturally-occurring compounds including saponins and flavonoids isolated from ethnopharmacologically well-known plants such as ginseng, cordyceps, and *Artemisia asiatica* are regarded as one of anti-cancer remedy candidates [2,3,4,5,6,7].

**Figure 1:**
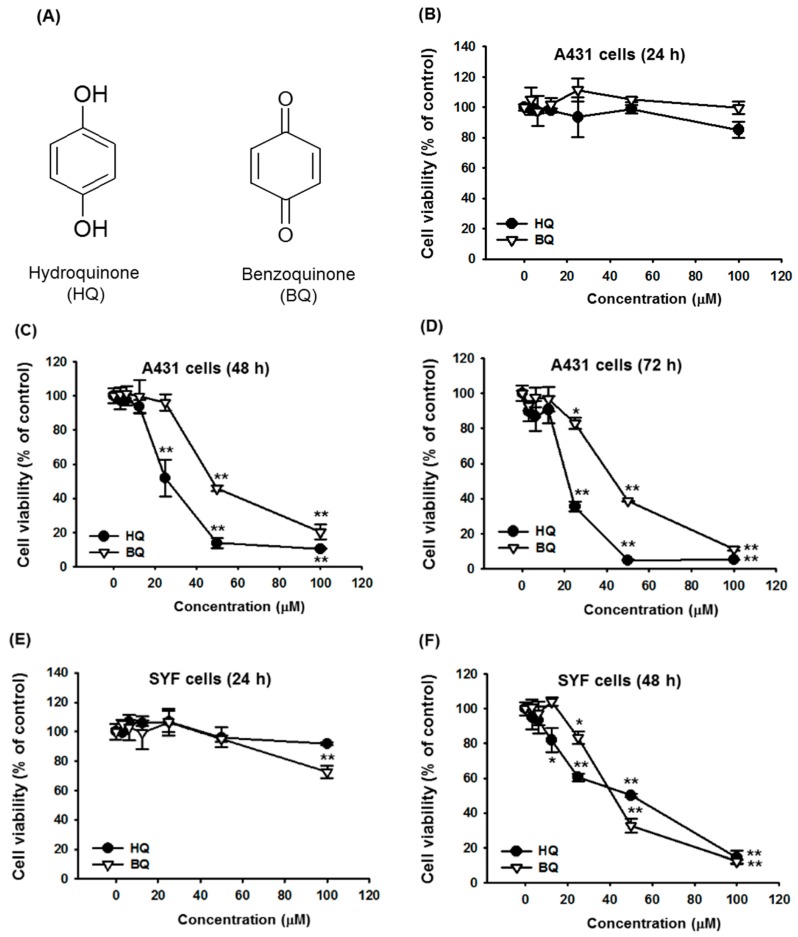

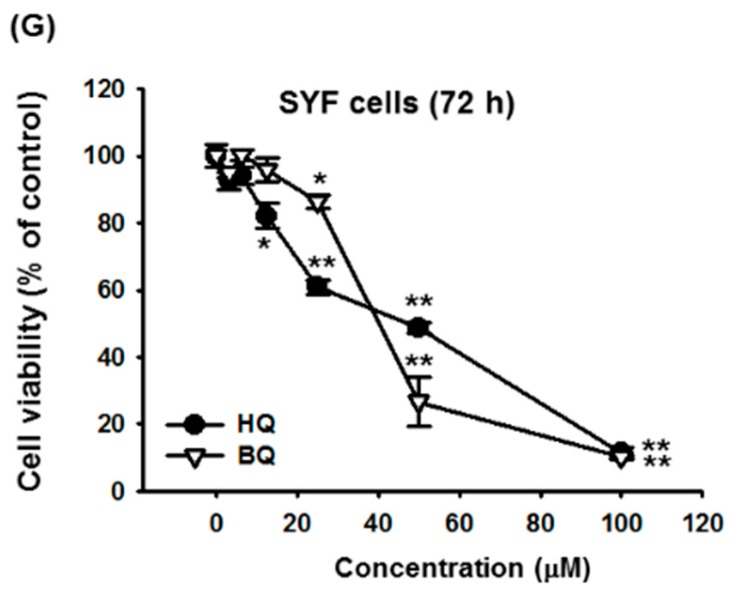
Effect of HQ on the proliferation of A431 and SYF cells. (**A**) Chemical structures of HQ and BQ. (**B**–**G**) A431 cells (1 × 10^6^ cells/mL) were treated with the indicated doses of either HQ or BQ for (**B**) 24 h, (**C**) 48 h, and (**D**) 72 h. SYF cells (1 × 10^6^ cells/mL) were treated with the indicated doses of either HQ or BQ for (**E**) 24 h, (**F**) 48 h, and (**G**) 72 h. Cell viability was determined via MTT assay. * *p* < 0.05 and ** *p* < 0.01 compared to normal.

Hydroquinone (HQ, 1,4-benzenediol, Figure 1A) is an aromatic organic compound that is a derivative of benzene, a type of phenol and a major constituent of several beverages, including beer, tea, and coffee [8,9]. HQ has been reported to exhibit various biological functions, such as anti-oxidative [10,11], neuroprotective [12,13], and immunomodulatory activities [14,15,16]. Moreover, several studies have demonstrated that HQ has anti-inflammatory activity. HQ inhibits the production and extracellular release of inflammatory mediators, such as nitric oxide (NO) and prostaglandin E_2_ (PGE_2_) and has alleviated symptoms of hepatitis in a mouse model [17]. In addition, HQ can suppress the expression of pro-inflammatory cytokines, including tumor necrosis factor-alpha (TNF-α), interleukin (IL)-1β, and IL-6 by targeting various molecules in the nuclear factor-κ B (NF-κB) signaling pathway in macrophages [18,19,20,21,22]. 

Despite several successful studies reporting the effects of HQ on various biological activities, the inhibitory activity of HQ on the pathogenesis of cancers is not well understood, and therefore needs to be investigated thoroughly. In this study, in vitro and in vivo anti-cancer activities of HQ were investigated using various cancer cells and tumor-bearing mouse models.

## 2. Results and Discussion

Although HQ has been demonstrated to have various biological functions, such as anti-oxidative, neuroprotective, immunomodulatory, and anti-inflammatory activities, its anti-cancer activity has not been thoroughly investigated. Therefore, in this study, in vitro and in vivo anti-cancer activity of HQ was explored using various cancer cells and cancer-bearing mouse models.

We first investigated the anti-cancer activity of HQ in vitro using cancer cell lines. A431 human squamous carcinoma cells were treated with escalating doses of HQ for 24, 48, and 72 h, and cell death caused by HQ was then evaluated. Treatment of HQ for 24 h did not induce the cell death of A431 cells (Figure 1B), whereas, 12.5 μM HQ induced dramatic death of A431 cells at 48 (Figure 1C) and 72 h (Figure 1D) in a dose-dependent manner. Similarly to HQ, BQ belongs to a quinone group and has been demonstrated to exhibit anti-cancer activity [23,24]. Therefore, we compared the anti-cancer activity of HQ on A431 cells with that of BQ. The cytotoxic effect of HQ on A431 cells was much higher than that of BQ (Figure 1C,D). We also examined the cytotoxic effect of HQ on SYF mouse embryonic fibroblast cells. In accordance with the results of A431 cells (Figure 1B–D), HQ did not induce the death of SYF cells at 24 h (Figure 1E). However, HQ exhibited a significant cytotoxic effect on SYF cells at 48 (Figure 1F) and 72 h (Figure 1G) in a dose-dependent manner. The cytotoxic effect of HQ on SYF cells was also compared with that of BQ. HQ exhibited a significantly higher cytotoxic effect on SYF cells compared to BQ except for at a dose of 50 μM (Figure 1F,G). These results indicate that HQ has stronger cytotoxic effects on cancer cells at lower doses compared to BQ, which is an anti-cancer agent of the same quinone group, indicating the potential of HQ as a new and better anti-cancer drug, as summarized in Table 1. Similarly, we also observed 2-fold higher inhibitory activity of HQ than that of BQ under NO release conditions from lipopolysaccharide-stimulated RAW264.7 cells [20], implying that HQ will show better pharmacological activities than BQ toward other biological or disease conditions.

**Figure 2:**
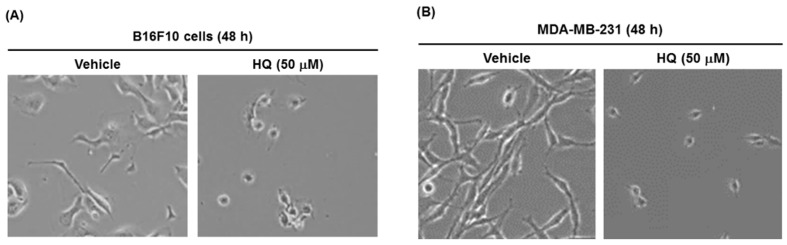
Effect of HQ on the morphological change of B16F10 and MDA-MB-231 cells (**A**,**B**) B16F10 cells and MDA-MB-231 cells were treated with either vehicle or HQ (50 μM) for 48 h, and cell morphologies were photographed.

To assess the HQ-induced cytotoxicity of cancer cells more directly, the in vitro anti-cancer activity of HQ was further investigated by observing morphological changes in cancer cells. In addition, morphological changes were evaluated in different types of cancer cells treated with HQ. B16F10 mouse melanoma cells were treated with HQ (50 μM) for 48 h, and then the cytotoxic effect of HQ on these cells was evaluated by observing their morphological changes under the microscope. HQ dramatically induced the shrinkage and death of B16F10 cells compared to vehicle-treated control cells (Figure 2A). We also applied this experimental approach to MDA-MB-231 human breast cancer cells. Similarly, HQ markedly induced the shrinkage and death of MDA-MB-231 cells compared to vehicle-treated control cells (Figure 2B). Combined with the results shown in Figure 1, these results suggest that HQ exhibits effective in vitro anti-cancer activity for various types of cancer, not just for certain types of cancer, indicating that HQ may be used as an anti-cancer drug for a broad spectrum of cancer types.

**Figure 3:**
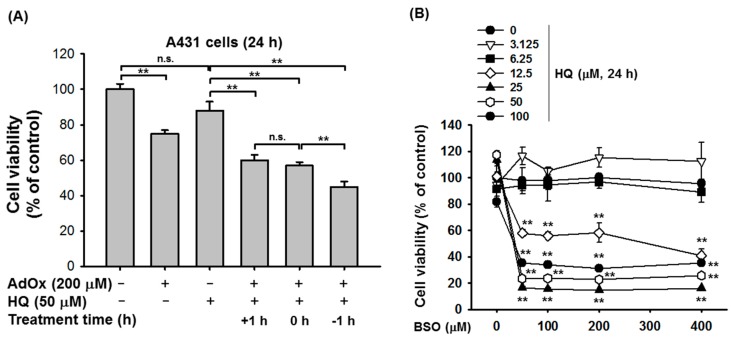
*S*ynergistic effect of HQ on the proliferation of A431 cells under AdOx- or BSO-treated conditions. (**A**) A431 cells were treated with HQ (50 μM) and AdOx (200 μM) for 24 h, and cell viability was determined via MTT assay. +1 h, HQ treatment 1 h after AdOx treatment; 0 h, HQ and AdOx treatment at the same time; and −1 h, HQ treatment 1 h before AdOx treatment. (**B**) A431 cells were treated with the indicated doses of HQ and BSO for 24 h, and cell viability was determined via MTT assay. n.s., not significant; ** *p* < 0.01 compared to the normal or control.

We next investigated in vitro synergistic anti-cancer activity of HQ with different anti-cancer agents. AdOx, an inhibitor of S-adenosylhomocysteine hydrolase, is used to study the role of S-adenosylhomocysteine hydrolase in adenosine-induced apoptosis and related S-adenosylhomocysteine-regulated processes. Interestingly, AdOx was reported to have anti-cancer activity [25,26]; therefore, the synergistic anti-cancer activity of HQ was examined with AdOx in cancer cells. For testing synergistic activity, we chose 200 and 50 μM of AdOx and HQ showing 10 to 25% of inhibitory activity after establishing dose-dependent inhibitory profiles. A431 cells were treated with HQ (50 μM for 24 h) 1 h before (−1 h), at the same time of (0 h), and 1 h after (+1 h) AdOx (200 μM) treatment. In accordance with the results shown in Figure 1B, HQ alone did not induce A431 cell death, however, co-treatment of HQ and AdOx significantly induced A431 cell death (Figure 3A). Interestingly, treatment of HQ 1 h prior to AdOx treatment (−1 h) had the highest cytotoxic effect on A431 cells compared to the 0 h or +1 h AdOx-treated groups (Figure 3A). These results signify that the time schedule for the administration of anti-cancer drugs may be critical for cancer patients receiving combination therapy. The in vitro synergistic anti-cancer activity of HQ was further investigated with BSO. Since sulfhydryl group (SH-) of cysteine in target proteins (Akt and Src) plays a critical role in mediating pharmacological activity of HQ [20,27], it was assumed that cellular level of reduced glutathione (GSH) could be important in controlling HQ’s activity. BSO was reported to block cellular resistance to chemotherapy by inhibiting γ-glutamylcysteine synthetase, a key enzyme in glutathione biosynthesis which can regulate the level of glutathione, and used as an adjunct with chemotherapy in the treatment of cancer [28,29,30]. Therefore, we employed BSO to test our assumption as to whether combination can increase the anti-proliferative activity of HQ. A431 cells were treated with escalating doses of HQ and BSO for 24 h, and then cell death was evaluated. As expected, HQ significantly induced A431 cell death at doses from 12.5 to 100 μM with BSO at doses from 50 to 400 μM (Figure 3B). This result confirmed the results shown in Figure 1A, whereby treatment of A431 cells with HQ alone for 24 h did not induce cell death, however, HQ in combination with BSO, which also could not induce A431 cell death alone, induced A431 cell death synergistically (Figure 3B). Interestingly, the synergistic anti-cancer activity of HQ with BSO was comparable with doses of HQ from 25 to 100 μM and doses of BSO from 50 to 400 μM (Figure 3B), although HQ displayed better cytotoxicity than BSO. These results suggest that lower doses of HQ (25 μM) and BSO (50 μM) may be sufficient for in vitro synergistic anti-cancer activity. Moreover, combination of HQ with BSO seems to prevent a potential risk of HQ regarding mutagenesis and genotoxicity at its higher concentrations [31].

How HQ can block the proliferation of cancer cells is not fully understood in this study. However, several points led us to postulate that suppression of Akt might be a major mechanism by which HQ suppresses viability of A431 and SYF cells (Figure 1 and Table 1). The first point is that HQ blocked the proliferation of SYF cells, a cell without Src (Figure 1). Based on our previous report that HQ directly upregulated Src kinase activity [27], positively required for enhancing cancer cell proliferation [5,32], effect of HQ on the proliferation of SYF cells looked to be not related to Src. Indeed, proliferation of SYF cells was not increased but rather decreased by HQ (Figure 1). As the second point, Akt, a cell survival-regulatory enzyme, can be considered as target of HQ. Thus, it was previously confirmed that HQ is able to bind to Cys-310 residue in Akt and subsequently block the phosphorylation of Thr-308 in Akt [20], an essential step to activate nuclear factor (NF)-κB pathway and to manage cell survival [33], via activation of inhibitor of κB kinase (IKK) [34,35,36]. Since there is lack of a direct evidence on the involvement of Akt as the target of HQ in this study, further detailed experiments will be continued to verify this possibility. In addition, there is another possibility that HQ can deplete cellular level of reduced glutathione by formation of HQ-reduced glutathione adduct via thiolation. Because glutathione is important molecule for protection of cells from oxidative stress, HQ-treated cells with depleted glutathione condition could be sensitive to oxidative stress inducing cytotoxicity of cancer cells. In terms of oxidative stress, we will also further confirm this possibility.

**Figure 4:**
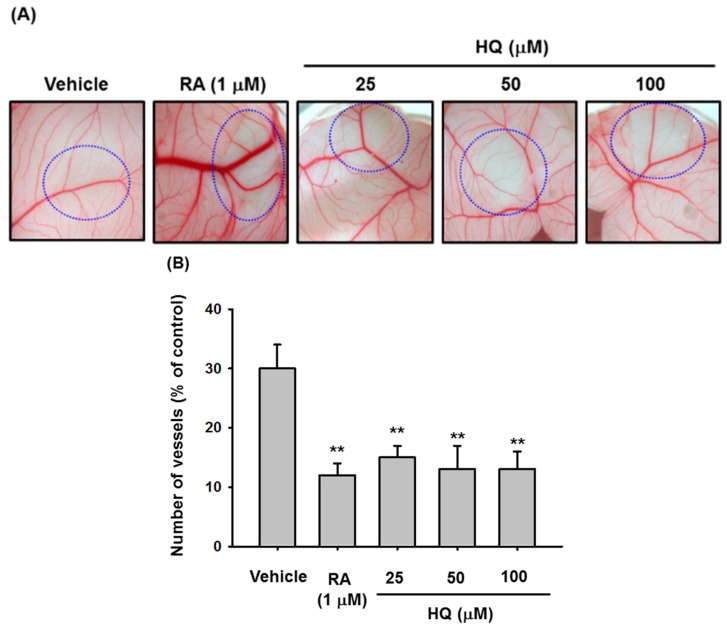
Effect of HQ on the blood vessel generation. (**A**,**B**) Fertilized chicken eggs were treated with either vehicle, RA, or the indicated doses of HQ (circle areas) at 37 °C for 3 days, and the vasculatures in the fertilized chicken eggs were photographed (**A**). Numbers of vessels at drug-treated area were counted by a counter (**B**). ** *p* < 0.01 compared to the vehicle control.

Angiogenesis is a biological process through which new blood vessels form from pre-existing blood vessels and one of the fundamental steps in the transition of tumors from a benign state to a malignant one. Therefore, we investigated the anti-angiogenic activity of HQ via CAM assay using fertilized chicken embryos. Fertilized chicken embryos were treated with either RA, an angiogenesis inhibitor [37], or escalating doses of HQ, and vasculatures in the fertilized chicken embryos were observed. Like RA, HQ markedly inhibited angiogenesis of the fertilized chicken embryos up to 70% (Figure 4A,B), indicating that HQ has anti-cancer activity related to angiogenesis inhibition.

**Figure 5:**
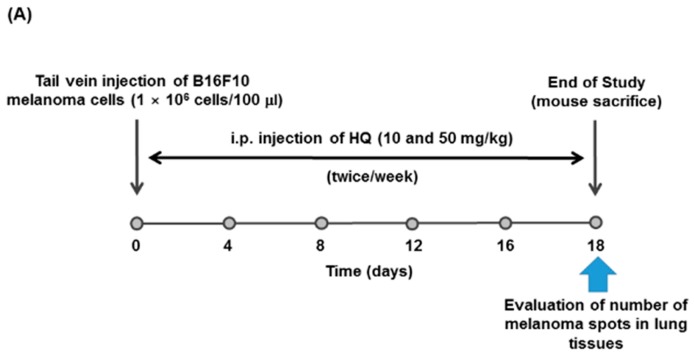

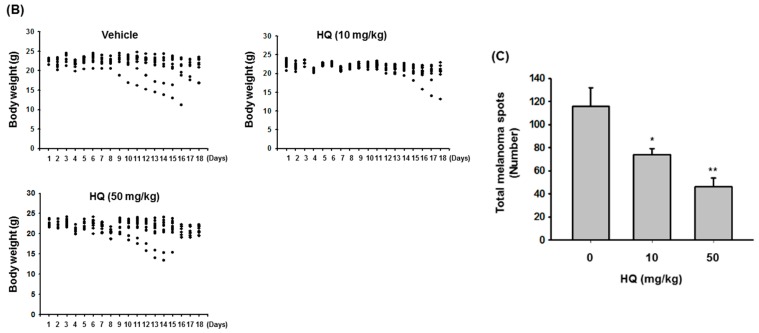
Effect of HQ on the lung metastasis of B16F10 melanoma. (**A**) Schematic diagram of in vivo lung metastasis model. (**B**) The body weight of B16F10 melanoma lung metastasis mice treated with either vehicle or the indicated doses of HQ was measured every day for 18 days. (**C**) B16F10 melanoma lung metastasis mice treated with either vehicle or the indicated doses of HQ were euthanized at day 19. After euthanasia, the lungs of the mice were excised and the total number of melanoma spots in the lungs of each group was counted and plotted. * *p* < 0.05 and ** *p* < 0.01 compared to the vehicle control.

We next investigated in vivo anti-cancer activity of HQ using cancer-bearing mouse models. A B16F10 melanoma lung metastasis model was generated by injecting B16F10 melanoma cells into the tail veins of mice, and the mice were injected with HQ (10 and 50 mg/kg) (Figure 5A). The body weight of the mice injected with vehicle or two different doses of HQ was comparable between groups, and significant loss of body weight was not observed in all groups during the experiment (Figure 5B). This indicates that HQ was well-tolerated by the mice in all groups and that HQ did not exhibit in vivo toxicity at the doses studied. Total melanoma spots metastasized in the mouse lungs were counted. As shown in Figure 5C, the total number of melanoma spots metastasized in the lungs of mice treated with HQ were dramatically reduced compared to those of vehicle-treated control mice in a dose-dependent manner.

**Figure 6:**
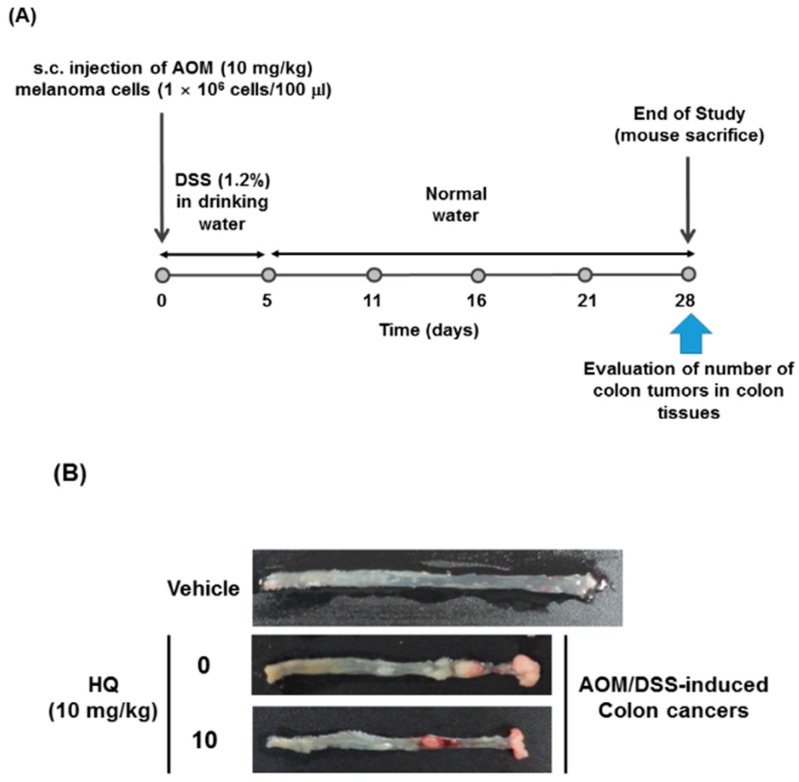

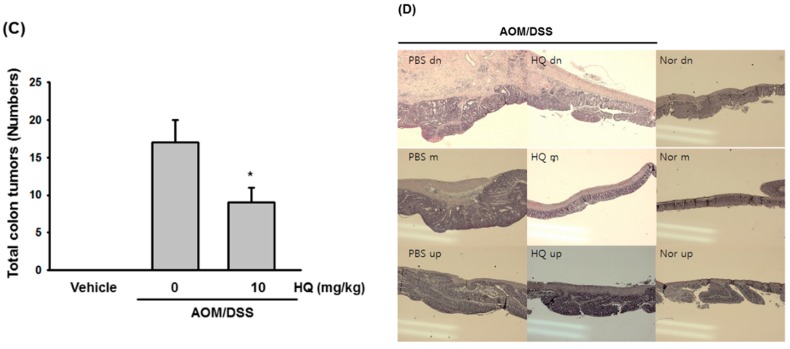
Effect of HQ on the generation of AOM/DSS-induced colon cancer in mice. (**A**) Schematic diagram of in vivo colitis-associated colon cancer model. (**B**) AOM/DSS-induced colon cancer mice were treated with either vehicle or HQ (10 mg/kg) twice a week for 4 weeks. After euthanasia, the colons excised from AOM/DSS-induced colon cancer mice were photographed and compared with that from normal mice treated with vehicle. (**C**) The total number of tumors generated in the colons of the mice were counted and plotted. (**D**) The three parts of the colons excised from these mice were stained with hematoxylin and eosin. dn, distal colon; m, middle colon; up, proximal colon. * *p* < 0.05 compared to the control.

Moreover, a colon cancer model was generated by injecting mice with azoxymethane (AOM) and dextran sodium sulfate (DSS), and the anti-cancer activity of HQ was further evaluated in vivo in this mouse cancer model (Figure 6A). HQ effectively prevented the generation of colon tumors in AOM/DSS-injected mice (Figure 6B). The total number of colon tumors decreased in AOM/DSS-injected mice treated with HQ compared to vehicle-treated control mice in a dose-dependent manner up to 50% (Figure 6C). In addition, histopathological analysis by hematoxylin and eosin staining of distal, middle, and proximal colons of the AOM/DSS-injected mice demonstrated that HQ markedly reduced the thickness of the colon tissues (Figure 6D). These results clearly indicate that in accordance with in vitro anti-cancer activity observations, HQ exhibits in vivo anti-cancer activity by reducing the generation and metastasis of tumors.

## 3. Materials and Methods

### 3.1. Materials

HQ, benzoquinone (BQ, Figure 1A), (3-4,5-dimethylthiazol-2-yl)-2,5-diphenyltetrazolium bromide (MTT), adenosine-2′,3′-dialdehyde (AdOx), buthionine sulfoximine (BSO), azoxymethane (AOM), dextran sodium sulfate (DSS), retinoic acid (RA), hematoxylin, and eosin were purchased from Sigma-Aldrich (St. Louis, MO, USA). Fetal bovine serum (FBS), phosphate buffered saline (PBS), and Dulbecco’s modified Eagle’s medium (DMEM) were purchased from GIBCO (Grand Island, NY, USA). A chick chorioallantoic membrane (CAM) assay kit was purchased from Thermo Fisher Scientific (Waltham, MA, USA).

### 3.2. Animal

C57BL/6 mice (male, 6–8 weeks old) were purchased from Daehan Biolink (Osong, Korea). The mice were housed in plastic cages (5 mice/cage) at 22 ± 2 °C and 55 ± 5% humidity on 12/12-h light/dark cycles, and given water and a pelleted diet (Samyang, Daejeon, Korea) *ad libitum*. All studies using the mice were conducted in accordance with the guideline of the Institutional Animal Care and Use of Laboratory Animal, published by the US National Institutes of Health (NIH, Bethesda, Rockville, MD, USA). The protocol was approved by the ethics committe of Sungkyunkwan University (Approval NO: SKKUBBI 12–6–1, 15 December 2012).

### 3.3. Cell Culture

A431, a human squamous carcinoma cell line; SYF, mouse embryonic fibroblasts deficient in c-Src, Yes, and Fyn; B16F10, a mouse melanoma cell line; and MDA-MB-231, a human breast cancer cell line were purchased from the American Type Culture Collection (Manassas, VA, USA). The cells were cultured in DMEM supplemented with 10% FBS, 100 mg/mL streptomycin, 100 U/mL penicillin, and 2 mM L-glutamine at 37 °C in a 5% CO_2_ humidified incubator. The cells were subcultured with fresh culture media two times a week.

### 3.4. Cell Viability Assay

A431 and SYF cells were treated with the indicated doses of HQ, BQ, AdOx, and BSO for 24, 28, and 72 h and the cell viability was determined by conventional MTT assay as previously described [38]. Briefly, 100 μL of cells in culture media were mixed with 10 μL of MTT solution (10 mg/mL in PBS, pH 7.4) followed by incubation at 37 °C for 4 h. Then 15% sodium dodecyl sulfate solution was added to the mixture, and the mixture was further incubated at 37 °C for 24 h. The OD was determined at 570 nm using a Spectramax 250 microplate reader. B16F10 cells and MDA-MB-231 cells were treated with HQ (50 μM) for 48 h. Cell morphologies were compared with vehicle-treated control cells and cell viability was determined from photographs taken under an optical microscope.

### 3.5. CAM Assay in Fertilized Chicken Eggs

CAM assay was performed as previously described [39] with slight modification. Briefly, fertilized chicken eggs were incubated at 37 °C with 60% humidity, and small windows were generated on the top of the eggshells at day 3. After the fertilized chicken embryo was treated with either RA (1 μM) or the indicated doses of HQ, the windows were resealed with adhesive tape and the fertilized chicken embryos were incubated at 37 °C for 3 days. Vasculatures of the fertilized chicken embryos were photographed the following day.

### 3.6. B16F10 Melanoma Lung Metastasis Model

A pulmonary metastasis model using B16F10 melanoma cells was generated in mice as previously described [40]. B16F10 cells were suspended in PBS (1 × 10^6^ cells/100 μL), and 1 × 10^6^ cells were intravenously injected into the tail veins of C57BL/6 mice (*n* = 7/group). After the B16F10 cell injection, the mice were intraperitoneally injected with either vehicle or HQ (10 and 50 mg/kg) twice a week for 18 days. Body weight of the mice was measured every day for 18 days. After euthanasia at day 18, the total number of melanoma spots metastasized in the lung tissue in each group of mice were counted.

### 3.7. AOM/DSS-Induced Colon Cancer Model

Colon cancer was generated in mice by injecting AOM and DSS as previously described [41,42]. C57BL/6 mice (*n* = 7/group) were subcutaneously injected with AOM (10 mg/kg) and one week later, the mice were given drinking water containing 1.2% (*w*/*v*) of DSS for 5 days. At day 6, the DSS-containing drinking water was replaced by normal drinking water, and the mice were intraperitoneally injected with either vehicle or HQ (10 mg/kg) twice a week for 4 weeks. After euthanasia at the end of the study, the colon tissues in each group of mice were photographed, and the total number of colon tumors in each group of mice were counted.

### 3.8. Hematoxylin and Eosin Staining

After euthanasia at the end of the study, colon tissues of normal and AOM/DSS-induced colon cancer mice injected with either vehicle or HQ were excised, and sections of the samples were prepared by fixing the colon tissue in 10% buffered formaldehyde solution and embedding in paraffin. Sections of colon tissues were stained with hematoxylin and eosin to observe pathological changes.

### 3.9. Statistical Analysis

All results presented in this study are expressed as mean ± SD from at least three independent experiments. For statistical analysis, the data were compared by an analysis of variance (ANOVA) or Scheffe’s post-hoc test and the Kruskal-Wallis or Mann-Whitney test. A *p* value less than 0.05 was considered statistically significant. All statistical analyses were conducted using SPSS software (SPSS Inc., Chicago, IL, USA).

## 4. Conclusions

**Figure 7:**
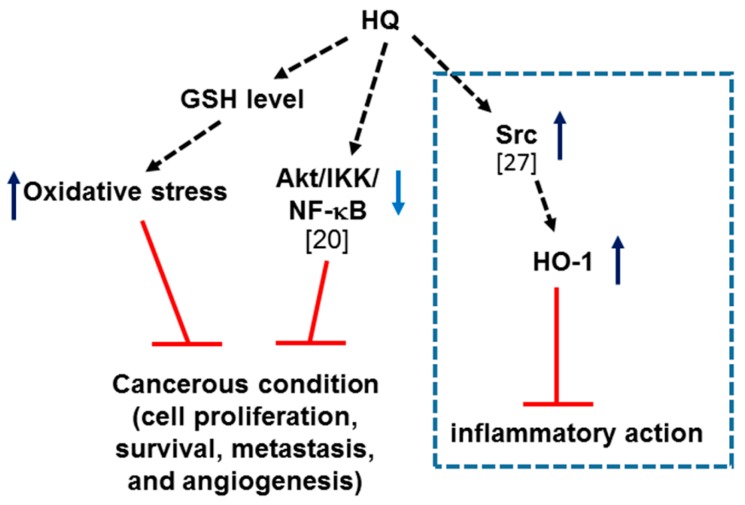
Putative inhibitory pathway of HQ-mediated anti-cancer activity. ↑ (ateneo blue): stimulation; ↓ (blue): inhibition; T (red): suppression of target biological responses.

In this study, we demonstrated the anti-cancer activity of HQ in vitro and in vivo using cancer cells and mouse cancer models as summarized in Figure 7. Although HQ was found to activate oncogenic Src linked to the upregulation of hemeoxygenase-1, an anti-oxidative protein [27], this compound rather induced cancer cell death alone and synergistically with other anti-cancer agents and also suppressed angiogenesis. Moreover, HQ prevented lung metastasis of melanoma cells without toxicity and formation of colon tumors in mice. In conclusion, these results suggest that HQ has anti-cancer activity in vitro and in vivo and has the potential to be developed into a promising drug for various types of cancer. However, to develop safer anti-cancer drugs with HQ, further deep studies including mutation of DNA and chromosomal rearrangement will be followed in the next project, since it is known that HQ is a potential genotoxicity-inducing drug [31]. To avoid genotoxicity, moreover, combination preparation with half or lower dose of HQ can be also tested. In addition, because we have previously reported several HQ derivatives (JS-III-49, -69, -73, -81, -87 and -89) with para form of hydroxyl groups still showing strong pharmacological activities [20,27], we are currently evaluating anti-cancer activities of these derivatives using in vivo models.

## Figures and Tables

**Table 1:** Inhibitory activities (IC_50_ values) of HQ and BQ.

Compound	IC_50_ Value (μM) at 72 h	
	A431 Cells	SYF Cells
HQ	23.3	37.5
BQ	54.1	52.3
